# Re-evaluating the link between internet use during pregnancy and low birth weight in light of maternal mental health

**DOI:** 10.1265/ehpm.25-00191

**Published:** 2025-08-06

**Authors:** Nagahide Takahashi, Akemi Okumura, Chika Kubota, Kenji J. Tsuchiya

**Affiliations:** 1 Department of Developmental Disorders, National Center of Neurology and Psychiatry, Kodaira, Japan; 2 Research Center for Child Mental Development, Hamamatsu University School of Medicine, Japan; 3 United Graduate School of Child Development, Osaka University, Japan; 4 Integrated Center for Women’s Health, National Center for Child Health and Development, Tokyo, Japan

**Keywords:** Internet use, Pregnancy, Low birth weight, Mental disorder, ADHD, Depression, Sleep disorders

To the Editor,

The recent study by Sakakihara et al. [1] reporting an association between prolonged internet use during pregnancy and low birth weight (LBW) (adjusted OR: 2.16, 95% CI: 1.13–4.13) raises timely concerns regarding digital behavior and perinatal outcomes. The topic is both novel and increasingly relevant, given the ubiquitous presence of screen-based technologies in daily life and the evolving understanding of their impact on maternal-child health. However, while the authors appropriately highlight methodological limitations related to geography, self-report, and health behavior data, we believe that their findings could be substantially enriched by considering maternal mental health as a central explanatory factor.

Sakakihara et al. do not assess depression, anxiety, ADHD, or sleep disorders—conditions highly prevalent in pregnancy and robustly linked to both elevated screen time and adverse birth outcomes. Perinatal depression affects up to 20% of pregnant individuals, while anxiety disorders occur in roughly 15–20% [2]. Adult ADHD, though underdiagnosed in obstetric settings, is estimated to affect approximately 2.5% of women of reproductive age globally, with some studies reporting rates as high as 4% in certain populations [3]. Sleep disturbances during pregnancy affect up to 80% of individuals [4]. These are not marginal conditions—they are central to understanding the behavioral and physiological milieu of pregnancy. Each of these conditions is independently associated with poor birth outcomes, including LBW, and with increased digital engagement. Their omission from the analytic framework not only weakens causal inference but also misses an opportunity to interpret screen time as a marker of psychosocial vulnerability.

For instance, individuals with ADHD often experience disrupted circadian rhythms, impulsivity, and a need for stimulation—patterns that can lead to late-night screen use [5]. Similarly, individuals with perinatal depression may retreat from physical activities and social interaction, turning instead to digital platforms for support or escape [6]. These behaviors, while labeled as problematic in the context of internet use, may reflect adaptive efforts to cope with mental distress. The same behavior—frequent internet use—may carry different meanings depending on the individual’s underlying mental health. Recognizing this complexity is essential for appropriate interpretation of observational findings.

Prior research has also demonstrated that ADHD increases the risk of adverse birth outcomes. In a recent international cohort, ADHD symptoms were associated with increased rates of both preterm birth (OR = 1.28, 95% CI = 1.19–1.37) and LBW (OR = 1.30, 95% CI = 1.17–1.43) [7]. ADHD has also been linked to behavioral patterns such as poor adherence to prenatal care, irregular eating habits, and sleep disturbances—all of which may impact fetal development. These findings raise the possibility that maternal ADHD could underlie both increased internet use and the risk of LBW observed in Sakakihara et al.’s study. Interestingly, the 4% prevalence of heavy internet use reported by Sakakihara et al. aligns with the upper range of adult ADHD prevalence estimates among women of reproductive age (approximately 2.5–4%). While this overlap does not establish causality, it supports the hypothesis that ADHD-related traits—such as impulsivity, sleep disruption, and executive dysfunction—may underlie both elevated screen engagement and susceptibility to adverse birth outcomes. This behavioral convergence invites further exploration of shared underlying mechanisms.

A similar line of evidence applies to maternal depression and anxiety, which not only co-occur with elevated screen use but are themselves associated with higher risk of LBW. Meta-analytic findings suggest an odds ratio of 1.84 (95% CI = 1.34–2.53) for LBW in individuals with prenatal depression. For anxiety, odds ratios are slightly lower but remain significant [8]. The causal mechanisms may involve alterations in neuroendocrine function, inflammation, and reduced engagement in prenatal health behaviors. Given that internet use may serve a regulatory function—offering distraction, support, or reassurance—it is essential to consider whether it acts not as a cause of risk, but as a coping strategy indicative of underlying mental distress.

Sleep disturbances further complicate this picture. Not only are they common during pregnancy, but they also serve as both a potential confounder and mediator in the relationship between internet use and LBW. Poor sleep may prompt digital engagement as a coping mechanism [9], which is then misinterpreted as a primary risk factor. Conversely, late-night internet use may worsen sleep, thereby indirectly affecting birth outcomes. Without time-of-day usage data or validated sleep assessments, it is difficult to determine the directionality of these relationships. Sakakihara et al. rightly note the possibility that screen time may interfere with sleep; we propose that poor sleep may also drive screen time. Testing both pathways empirically would strengthen the study’s contributions.

Additionally, the potential for genetic confounding must not be overlooked. Psychiatric disorders and LBW are both influenced by heritable factors [10]. Twin and sibling studies have demonstrated that familial vulnerability to psychiatric conditions can contribute to both behavioral patterns such as screen use and adverse birth outcomes. Without accounting for maternal psychiatric history or genetic liability, residual confounding remains a serious concern. Future studies may benefit from incorporating genetically informed designs or polygenic risk scores to clarify these associations.

We suggest that Sakakihara et al. or future investigators reanalyze or replicate this study with validated assessments of maternal mental health. This could include standardized tools such as the Edinburgh Postnatal Depression Scale (EPDS), the Generalized Anxiety Disorder 7-item scale (GAD-7), or the Adult ADHD Self-Report Scale (ASRS). Mediation analyses could test whether psychiatric symptoms partially or fully account for the association between screen time and LBW. Stratified analyses might clarify whether screen time poses greater risk in individuals with co-occurring psychological distress. Even qualitative data—asking pregnant participants about their reasons for digital engagement—could yield important distinctions between harmful overuse and functional coping.

Furthermore, Sakakihara et al. raise the possibility that internet use displaces healthier behaviors such as balanced nutrition or routine prenatal visits. This is a meaningful hypothesis, but we would caution that such behavioral disruptions may themselves be driven by psychiatric symptoms. For instance, executive dysfunction in ADHD may impair scheduling and consistency, while depressive symptoms may reduce appetite or motivation. Targeting internet use as a behavioral endpoint without addressing these upstream mental health contributors may result in limited intervention efficacy.

Rather than discourage screen use universally, we argue that elevated digital engagement should be viewed as a potential clinical signal. In clinical practice, frequent screen use could prompt sensitive inquiry into the individual’s sleep, emotional wellbeing, and support systems. The question should not be “How much time are you online?” but “What role does this play in your day, and how are you feeling?” In this sense, screen time is not the problem—it is a window into deeper needs.

Digital behavior is multifaceted. Pregnant individuals use the internet for health information, communication with providers, social support, relaxation, and distraction. Aggregating all these uses into a single “risk” category obscures their meaning. We recommend that future studies categorize screen content and context, or use ecological momentary assessment to better understand moment-to-moment motivations. Sakakihara et al.’s dataset may even permit retrospective analysis along these lines, enriching its clinical relevance.

Finally, mental health variables should not be viewed only as confounders, but as moderators and mediators that shape how digital behavior relates to outcomes. The same screen use pattern may exert different effects depending on the user’s psychological state. This nuance is crucial in both research interpretation and public messaging. Pathologizing screen time in pregnancy without understanding its psychological underpinnings risks stigmatizing those who are already vulnerable.

We commend Sakakihara et al. for addressing a topic of growing importance. Their work opens new space for inquiry at the intersection of digital behavior and reproductive health. We believe that integrating maternal mental health more explicitly into future iterations of this research will improve causal modeling, reduce stigma, and support more nuanced clinical recommendations for pregnant individuals in an increasingly digital world.
